# 6-Benzylaminopurine Alleviates the Impact of Cu^2+^ Toxicity on Photosynthetic Performance of *Ricinus communis* L. Seedlings

**DOI:** 10.3390/ijms222413349

**Published:** 2021-12-12

**Authors:** Puthukkolli P. Sameena, Hazem M. Kalaji, Krystyna Żuk-Gołaszewska, Tomasz Horaczek, Edyta Sierka, Jos T. Puthur

**Affiliations:** 1 Plant Physiology and Biochemistry Division, Department of Botany, University of Calicut, Calicut University P.O., Malappuram 673635, Kerala, India; sameenapp2017@gmail.com; 2 Department of Plant Physiology, Institute of Biology, Warsaw University of Life Sciences SGGW, 02-776 Warsaw, Poland; hazem@kalaji.pl; 3 Department of Agrotechnology and Agribusiness, Faculty of Agriculture and Forestry, University of Warmia and Mazury in Olsztyn, ul. Oczapowskiego 8, 10-719 Olsztyn, Poland; kzg@uwm.edu.pl; 4 Institute of Technology and Life Sciences—National Research Institute, Falenty, Al. Hrabska 3, 05-090 Raszyn, Poland; t.horaczek@itp.edu.pl; 5 Institute of Biology, Biotechnology and Environmental Protection, Faculty of Natural Sciences, University of Silesia in Katowice, 28 Jagiellonska, 40-032 Katowice, Poland; edyta.sierka@us.edu.pl

**Keywords:** 6-benzylaminopurine, antioxidation, chlorophyll *a* fluorescence, cytokinin, membrane stability, kinetin, reactive oxygen species

## Abstract

Copper (Cu) is an essential element involved in various metabolic processes in plants, but at concentrations above the threshold level, it becomes a potential stress factor. The effects of two different cytokinins, kinetin (KIN) and 6-benzylaminopurine (BAP), on chlorophyll *a* fluorescence parameters, stomatal responses and antioxidation mechanisms in castor (*Ricinus communis* L.) under Cu^2+^ toxicity was investigated. *Ricinus communis* plants were exposed to 80 and 160 μM CuSO_4_ added to the growth medium. Foliar spraying of 15 μM KIN and BAP was carried out on these seedlings. The application of these cytokinins enhanced the tissue water status, chlorophyll contents, stomatal opening and photosynthetic efficiency in the castor plants subjected to Cu^2+^ stress. The fluorescence parameters, such as Fm, Fv/Fo, Sm, photochemical and non-photochemical quantum yields, energy absorbed, energy trapped and electron transport per cross-sections, were more efficiently modulated by BAP application than KIN under Cu^2+^ toxicity. There was also effective alleviation of reactive oxygen species by enzymatic and non-enzymatic antioxidation systems, reducing the membrane lipid peroxidation, which brought about a relative enhancement in the membrane stability index. Of the various treatments, 80 µM CuSO_4_ + BAP recorded the highest increase in photosynthetic efficiency compared to other cytokinin treatments. Therefore, it can be concluded that BAP could effectively alleviate the detrimental effects of Cu^2+^toxicity in cotyledonary leaves of *R. communis* by effectively modulating stomatal responses and antioxidation mechanisms, thereby enhancing the photosynthetic apparatus’ functioning.

## 1. Introduction

The environmental pollutants released by anthropogenic activities are threatening the existence of biological systems. Among the various pollutants, heavy metals are a serious environmental threat which affects the physiology and metabolism of plants [1]. Heavy metals such as cadmium (Cd), chromium (Cr), copper (Cu), iron (Fe), lead (Pb), nickel (Ni), and zinc (Zn) are common pollutants. The distribution of these metals is influenced by industrial effluents, mining, smelting, and various agricultural activities [2]. Copper (Cu) is an essential element with diverse functions in plant metabolism and development, including roles in photosynthesis and respiration. At concentrations above the threshold level in the growing medium, Cu becomes extremely toxic and causes damages to the DNA, alterations in cell membrane integrity, and interferes in enzyme activity [3].

Previous studies revealed that stress caused by Cu resulted in phytotoxic effects on physiology, metabolism, and molecular processes in plants, and the overproduction of reactive oxygen species (ROS) is one of the prime reasons for these changes [4,5,6,7]. Among these, photosynthesis is most seriously affected by heavy metal stressors [8]. In addition, heavy metals negatively affect the structure and functions of chloroplasts, reduce chlorophyll biosynthesis, and interfere with the activities of the Calvin cycle, which arise from the interaction of absorbed metals inside the photosynthetic apparatus [9,10]. The enhanced activities of antioxidant enzymes and various non-enzymatic antioxidants act as a photoprotective mechanism in plants exposed to heavy metal stress, which successfully scavenge the excess ROS molecules and thereby prevent ROS-mediated membrane damage in metallophytes [11,12]. 

*Ricinus communis* (castor oil plant) is a perennial plant with higher heavy metal accumulation potential. It can grow luxuriously in heavily metal contaminated soils and accumulate metal ions at exceedingly higher concentrations in the shoot [13]. Thus, it is often found along the roadsides and wastelands characterized by multiple abiotic stresses. Plants growing in metal-contaminated lands have developed defense strategies to cope with toxic metal ions, including phytostabilization, depositions in the cell wall and trichomes, localization in the vacuoles, and elevation of antioxidant systems [14]. Our previous studies have proved that cotyledonary leaves effectively buffer Cu-induced toxicity symptoms in the primary leaves of castor plants by enabling the Cu^2+^ bioaccumulation in the cotyledonary leaves and enhancing the synthesis of various metabolites necessary to counter Cu toxicity [15].

It was reported that endogenous cytokinins have a protective role in plants during heavy metal stress, which may be developed due to the direct influence of cytokinins in the modulation of the metallothionein-like gene (MT-L2) or by the indirect effect of cytokinin-mediated antioxidant activity [16]. In recent years, researchers have demonstrated that hormonal supplementation results in enhanced plant tolerance during various abiotic stress situations, such as heavy metals, drought, salinity, water, and temperature [17,18,19,20]. The application of hormones such as auxins, abscisic acid, cytokinins, and brassinosteroids to plants subjected to various stresses enhances antioxidant activity and regulation of endogenous hormone signaling [20]. Cytokinins play significant roles in the growth and development of plants and are significantly involved in plant stress resistance. They can slow down metal-induced leaf senescence by scavenging the reactive oxygen species [21]. According to the findings of Singh et al. [22], kinetin supplementation in tomato seedlings subjected to Cd stress significantly alleviated the metal toxicity by increasing the physiological functioning and detoxification capability via modulation of the ascorbate-glutathione cycle. Likewise, 6-benzylaminopurine (BAP) application along with abscisic acid in tomato plants reduced the uptake of cobalt (Co), its translocation, and bioaccumulation to shoots [23]. Our previous studies have proved that the exogenous application of cytokinins confers Cu^2+^ stress tolerance to *R. communis* seedlings via modulation of secondary metabolite production [24]. Therefore, it can be observed that cytokinin has an elicitation role in plant adaptation to heavy metal stress. In this context, the present work compares the ameliorative effects of exogenous kinetin (KIN) and 6-benzylaminopurine (BAP) on the photosynthetic processes, stomatal responses and related antioxidation mechanisms in the cotyledonary leaves of *R. communis* subjected to Cu^2+^ toxicity. Though there are some reports regarding the ameliorative effects of exogenously applied cytokinins such as zeatin, KIN, and BAP towards heavy metal stress, this is the first report on the comparative study of KIN- and BAP-mediated enhanced photosynthetic efficiency and associated antioxidation mechanisms in *R. communis* seedlings under Cu^2+^ toxicity.

## 2. Results

**Figure 1:**
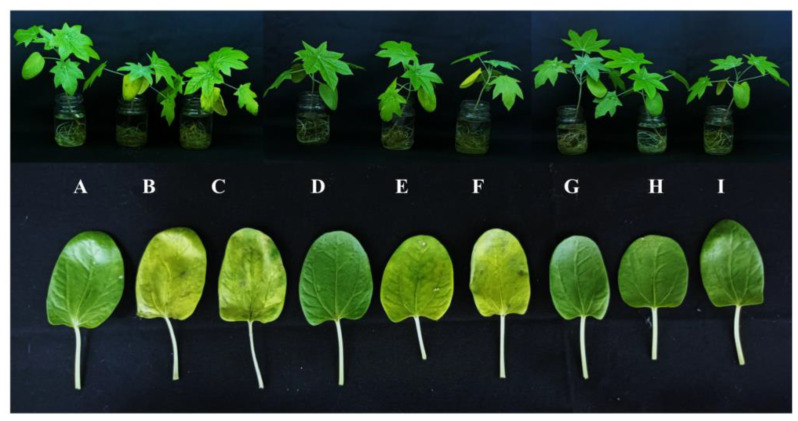
The phenotypic changes of cotyledonary leaves in *R. communis* after 6 day of exposure to CuSO_4_ (80 and 160 µM) and cytokinin (KIN and BAP) treatments in Hoagland’s nutrient medium. A: Control; B: 80 µM CuSO_4_; C: 160 µM CuSO_4_; D: KIN; E: 80 µM CuSO_4_+ KIN; F: 160 µM CuSO_4_+ KIN; G: BAP; H: 80 µM CuSO_4_+ BAP; and I: 160 µM CuSO_4_+ BAP.

The impact of KIN and BAP on the phenotype of *R. communis*, especially in the cotyledonary leaves exposed to Cu^2+^ toxicity, is represented in Figure 1. In this study, it was also observed that the application of cytokinins delayed the Cu^2+^ induced early abscission of cotyledonary leaves.

### 2.1. Effect of Cytokinins on Maintaining Tissue Water Status during Cu^2+^Toxicity

*Ricinus communis* treated with CuSO_4_ (80 and 160 µM) exhibited significant variations in the dry weight (*DW*)%, moisture content (*MC*)%, and relative water content (*RWC*) of cotyledonary leaves. The increase in *DW*% of cotyledonary leaves was 2.5- and 3.4-fold upon treatment with 80 and 160 µM CuSO_4_ concentrations, respectively, compared to the control. Application of KIN and BAP reduced the *DW*% of cotyledonary leaves in *R. communis* subjected to both concentrations of CuSO_4_ to a level similar to that of the control. Dry weight % was significantly reduced to the extent of 52–73% upon application of cytokinins along with CuSO_4_ as compared to those exposed to CuSO_4_alone (Table 1).

A significant reduction in *MC*% and *RWC* was observed in the cotyledonary leaves of *R. communis* subjected to Cu^2+^ toxicity. The reductions were to the extent of 27% and 68% in *MC*% and *RWC*, respectively, in cotyledonary leaves of *R. communis* on exposure to 160 µM CuSO_4_. In contrast, on the application of KIN and BAP along with CuSO_4_, the *MC*% and *RWC* of cotyledonary leaves were increased significantly. The enhancement in *MC*% and *RWC* was 39% and 181%, respectively, for the application of cytokinins along with 160 µM CuSO_4_ as compared to those plants exposed to CuSO_4_ alone (Table 1).

### 2.2. Effect of Cytokinins on Cu^2+^Induced Pigment Loss

Copper stress caused a significant reduction in total chlorophyll and carotenoid contents in cotyledonary leaves. The highest Cu concentration of the treatments (160 µM CuSO_4_) decreased the total chlorophyll and carotenoids contents by 39% and 58%, respectively, compared to the control. The application of KIN and BAP exogenously caused a significant increase in the pigment content compared to CuSO_4_ treatments alone. For the treatment of *R. communis* with 160 µM CuSO_4_ along with BAP, the total chlorophyll and carotenoid contents in the cotyledonary leaves were enhanced by 106% and 192%, respectively, as compared to the plants subjected to 160 µM CuSO_4_ alone (Table 2).

### 2.3. Effect of Cytokinins on Cu^2+^-Induced Reduction in Photosystem Activities

**Figure 2:**
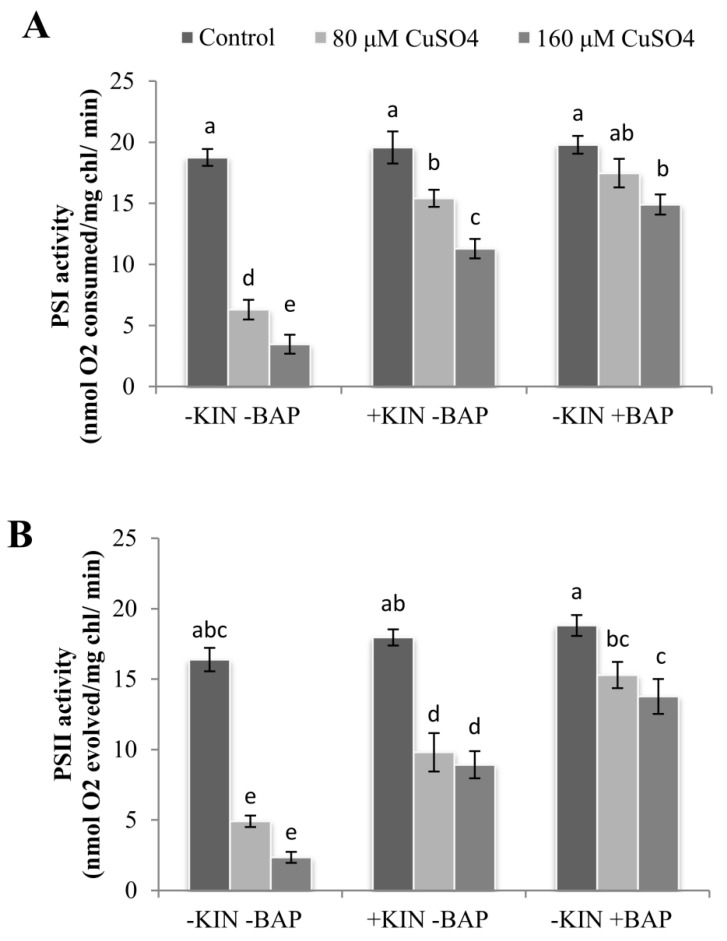
Photosystem (PS) I (**A**) and II (**B**) activities in the cotyledonary leaves of *R. communis* on 6 day of exposure to CuSO_4_ (80 and 160 µM) and cytokinin (KIN and BAP) treatments in Hoagland’s nutrient medium. Values are the mean ± SE of three independent experiments. Different alphabetical letters indicate significant differences between treatments according to one-way ANOVA (Duncan’s test *p* ≤ 0.05).

Similar to the reductions in photosynthetic pigments, photosystem I and II (PSI and PSII) activities were also reduced drastically on exposure to CuSO_4_. At 160 µM CuSO_4_, the reductions in PSI and PSII activities were 82% and 86%, respectively, compared to the control. On application of KIN and BAP, the activities of both PSI and PSII were increased dramatically, and this was most significant in BAP-treated cotyledonary leaves. In the plants treated with 160 µM CuSO_4_ along with KIN or BAP, the enhancement in PSI and PSII activities of cotyledonary leaves ranged between 3–4 and 4–6 fold, respectively, as compared to 160 µM CuSO_4_ alone (Figure 2).

### 2.4. Modulation of Chlorophyll Fluorescence Parameters by Cytokinin Application

**Figure 3:**
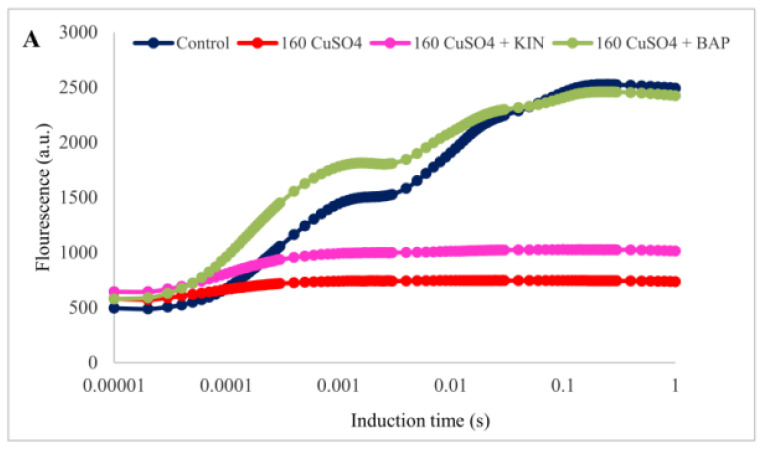

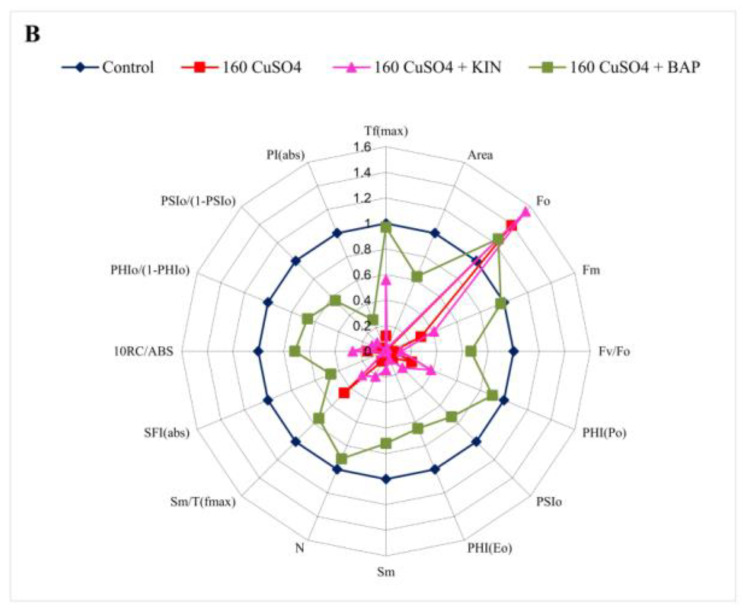
Fluorescence induction curve (**A**) and radar plot (**B**) in the cotyledonary leaves of R. communis for 4 day of exposure to CuSO_4_ (160 µM) and cytokinin (KIN and BAP) treatments in Hoagland’s nutrient medium.

The alteration in the light-dependent photosynthesis in the cotyledonary leaves of *R. communis* under Cu toxicity, along with the cytokinin applications, were evident in the chlorophyll fluorescence induction curves (Figure 3A). The fluorescence intensity represented on a logarithmic time scale starts with the initial fluorescence (Fo) at ‘O’ level passes through transitional fluorescences Fj and Fi at ‘J’ and ‘I’ levels, respectively, until attaining the point of maximum fluorescence (Fm) at ‘P’ level. This reveals the proper functioning of PSII. In the cotyledonary leaves of *R. communis*, when subjected to Cu^2+^ stress (160 μM CuSO_4_), the PSII efficiency was reduced drastically, as evidenced by the flattening of the fluorescence induction (OJIP) curve, especially at the J peak. However, for the application of BAP along with 160 μM CuSO_4_, the OJIP curve was restored to its standard shape, similar to that of the control. In contrast, KIN application, along with 160 μM CuSO_4_, could not restore the OJIP curve to its standard shape, and it was similar to the cotyledonary leaves treated with 160 μM CuSO_4_ alone.

In the radar plot representing various fluorescence parameters and performance indices, pronounced variations were observed in the cotyledonary leaves when the plants were treated with 160 μM CuSO_4_ compared to the control (Figure 3B). The fluorescence parameters such as Fm (maximal fluorescence), Tf(max) (time taken to reach Fm), Area (area over fluorescence induction curve), Fv/Fo (the efficiency of water splitting complex), and Sm (the relative pool size of plastoquinone) were drastically reduced (70–99%) in the cotyledonary leaves of *R. communis* subjected to 160 μM CuSO_4_ treatment. Plant photochemical performance (PI(abs)) and vitality indices (SFI(abs)) were significantly reduced to 99% on exposure to Cu^2+^ toxicity compared to the control. The yield parameters, including photochemical and non-photochemical quantum yields, exhibited prominent variations among the control and Cu^2+^ treated cotyledonary leaves. The values of PHI(Po), PHI(Eo), and PSIo were decreased to 78–98% in the cotyledonary leaves subjected to 160 μMCuSO_4_ as compared to the control. On application of BAP along with CuSO_4_, the photosynthetic efficiency was restored in the cotyledonary leaves, more or less similar to the control. In contrast, no significant variations were observed in cotyledonary leaves for KIN application along with CuSO_4_. Fm and Tf(max) values were reduced only negligibly, and at the same time Fo, Area, Fv/Fo, and Sm were reduced to a range of 24–33% in cotyledonary leaves of plants exposed to 160 μM CuSO_4_ along with BAP as compared to controls. The photochemical performance indices and yield parameters were also effectively modulated in the cotyledonary leaves for the application of BAP along with CuSO_4_ as compared to that of 160 μM CuSO_4_ alone (Figure 3B).

The energy flux parameters represent the various activities of the photosynthetic apparatus such as absorption, maximum trapping flux beyond Q_A_, electron transport, and dissipation of absorbed energy, expressed per excited cross-section (ABS/CSm, TRo/CSm, ETo/CSm, and DIo/CSm) and the specific activity in a single reaction center of PSII (ABS/RC, TRo/RC, ETo/RC, and DIo/RC) (Table 3). These parameters help to analyze the stepwise flow of energy through PSII at the level of cross-section (CS) as well as a single reaction center (RC). A significant reduction was observed in the number of photons absorbed (ABS/CSm), trapped energy (TRo/CSm), and electron transport (ETo/CSm) per cross-section in the cotyledonary leaves of *R. communis* subjected to 160 µM CuSO_4_ treatment. The reductions were 70%, 93% and 99%, respectively. However, in the case of a single reaction center, ETo/RC was drastically decreased (88%), but at the same time, ABS/RC (7-fold) and TRo/RC (52%) were significantly enhanced on exposure to 160 µM CuSO_4_ compared to the controls. In the case of dissipated energy (DIo/CSm and DIo/RC), a significant increase was recorded in cotyledonary leaves of plants subjected to 160 µM CuSO_4_ compared to controls, with an enormous hike observed in DIo/RC (26-fold).

The application of BAP efficiently modulated the changes in phenomenological energy parameters caused by Cu^2+^ stress. In contrast, KIN application along with CuSO_4_ did not impart any significant variations in PSII photochemistry, as evidenced by the variations in the OJIP kinetics and energy parameters as compared to those exposed to CuSO_4_ alone. For the application of BAP along with CuSO_4_, the energy parameters such as ABS/CSm, TRo/CSm, ETo/CSm, and ETo/RC were enhanced in cotyledonary leaves of plants as compared to that of CuSO_4_ treatment alone, and it was almost on par with the respective controls. In the case of ABS/RC and TRo/RC, the observed reductions were 79% and 16% compared to CuSO_4_ treatment alone (Table 3).

### 2.5. Cytokinin Mediated Stomatal Responses

**Figure 4:**
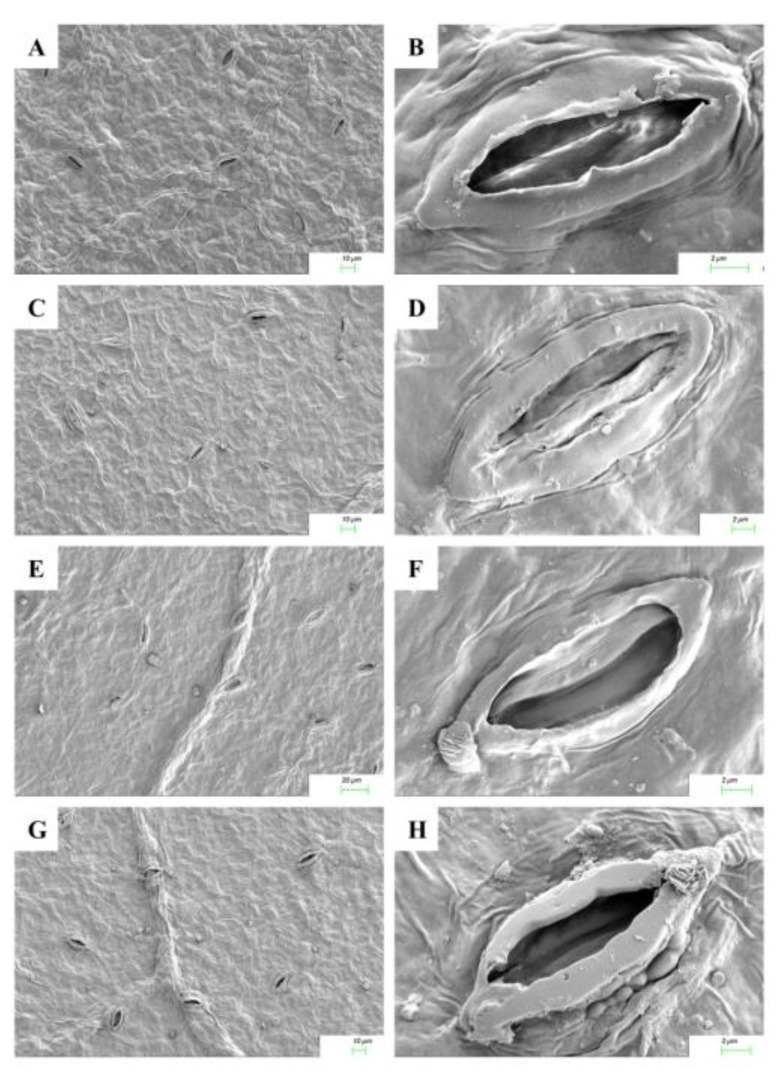
Scanning electron micrographs of adaxial leaf surfaces and stomata of the cotyledonary leaves of *R. communis* after 6 day of exposure to CuSO_4_ (160 µM) and cytokinin (KIN and BAP) treatments in Hoagland’s nutrient medium.(**A**,**B**): Control; (**C**,**D**): 160 µM CuSO_4_; (**E**,**F**): 160 µM CuSO_4_ + KIN; (**G**,**H**): 160 µM CuSO_4_ + BAP.

**Figure 5:**
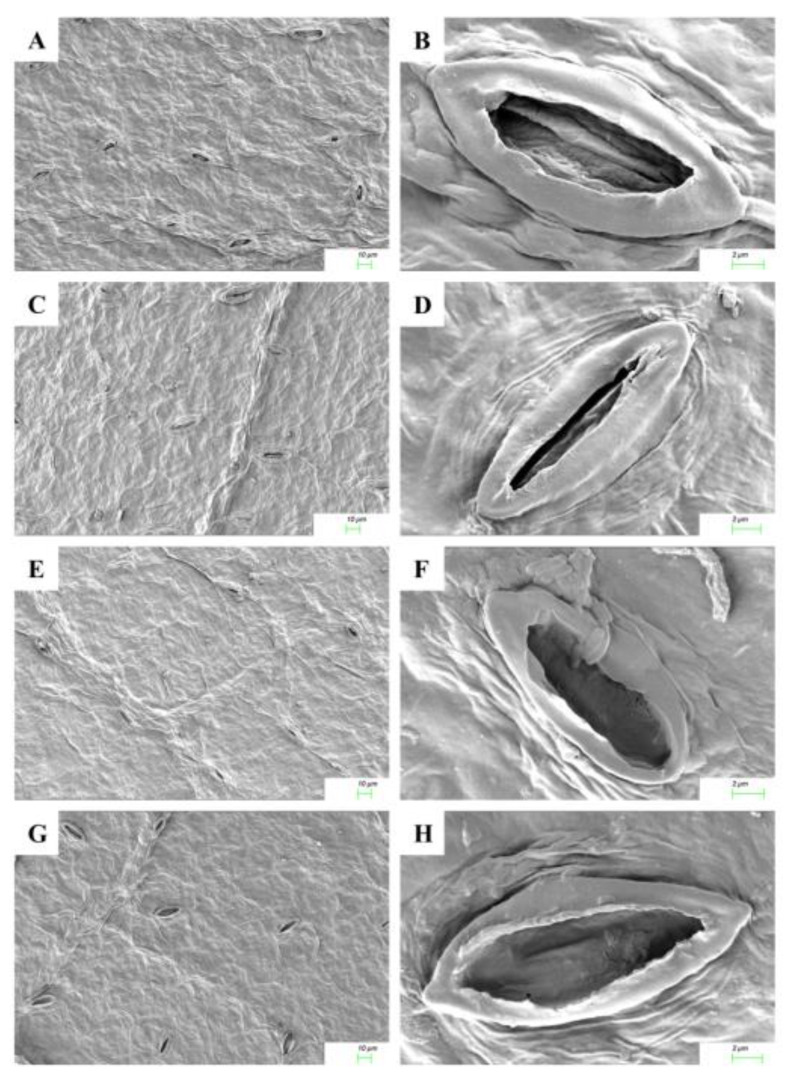
Scanning electron micrographs of abaxial leaf surfaces and stomata of the cotyledonary leaves of *R. communis* after 6 day of exposure to CuSO_4_ (160 µM) and cytokinin (KIN and BAP) treatments in Hoagland’s nutrient medium. (**A**,**B**): Control; (**C**,**D**): 160 µM CuSO_4_; (**E**,**F**): 160 µM CuSO_4_ + KIN; (**G**,**H**): 160 µM CuSO_4_ + BAP.

The responses of stomatal opening on exposure to Cu^2+^ stress and cytokinin application in adaxial and abaxial leaf surfaces are shown in Figure 4 and Figure 5. The micromorphological characters such as the number of stomata per unit area in the cotyledonary leaves of *R. communis* were significantly reduced in both adaxial and abaxial surfaces during CuSO_4_ treatment as compared to controls. On application of KIN and BAP, the frequency of stomata was enhanced in both adaxial and abaxial surfaces. The adaxial stomata were completely closed, whereas the abaxial stomata were partially closed during Cu^2+^ stress, as compared to the respective controls. However, upon application of cytokinins, the stomata opened on both adaxial and abaxial surfaces. In contrast to the KIN application, the stomatal opening was more prominent with the application of BAP (Figure 4 and Figure 5).

### 2.6. Cytokinin Induced Decline in ROS and MDA Accumulation

**Figure 6:**
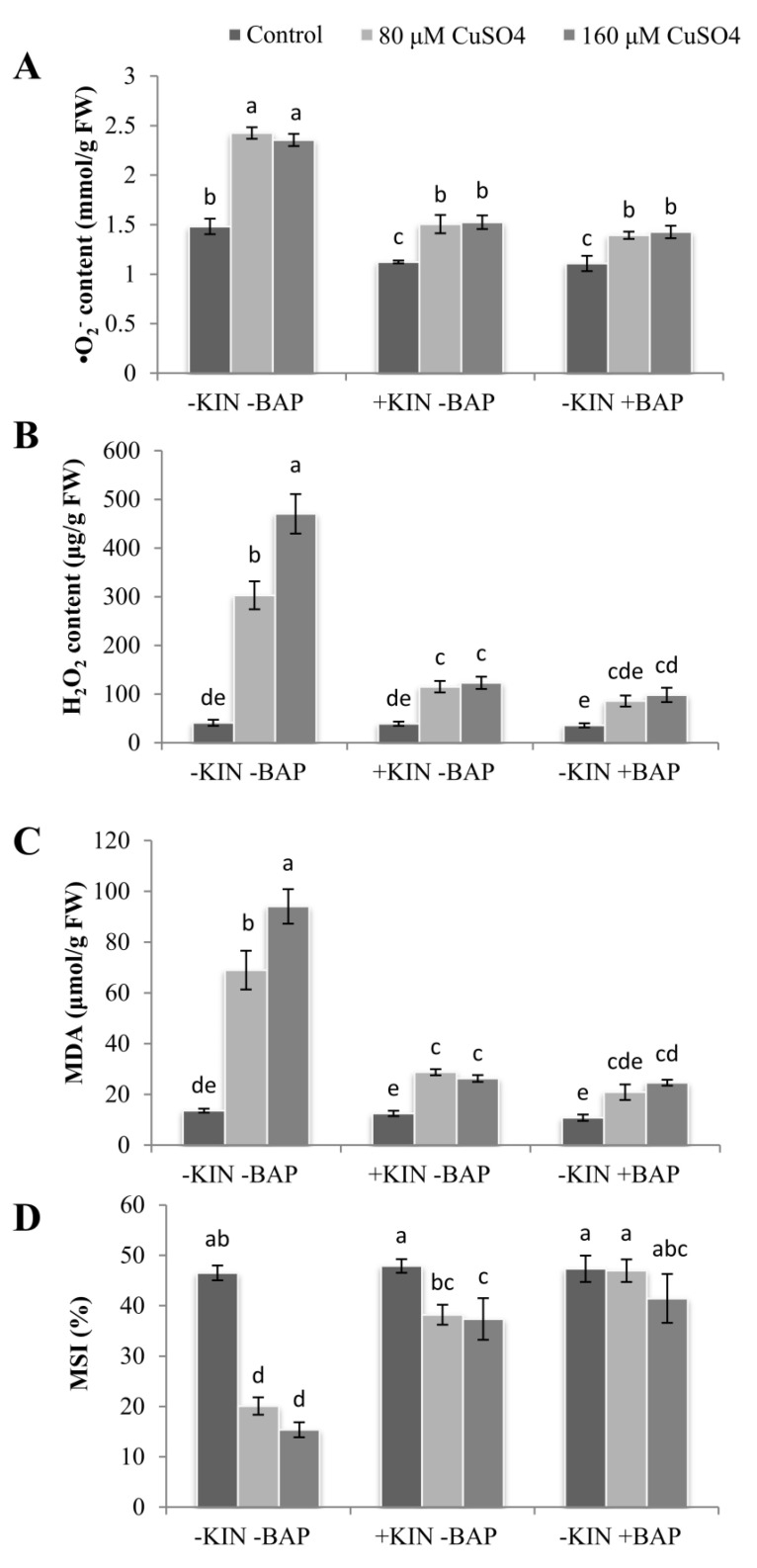
Superoxide (**A**), hydrogen peroxide (**B**), MDA content (**C**) and membrane stability index (**D**) in the cotyledonary leaves of *R. communis* after 6 day of exposure to CuSO_4_ (80 and 160 µM) and cytokinin (KIN and BAP) treatments in Hoagland’s nutrient medium. Values are the mean ± SE of three independent experiments. Different alphabetical letters indicate significant differences between treatments according to one-way ANOVA (Duncan’s test *p* ≤ 0.05).

The accumulation of ROS molecules was significantly increased in the cotyledonary leaves of plants subjected to CuSO_4_ treatments compared to the cotyledonary leaves of control plants. The increase in superoxide (O_2_^−^) content was 88–93% on exposure to 80 and 160 µM CuSO_4_ compared to controls.On application of KIN or BAP along with 160 µM CuSO_4_, O_2_^−^ content was significantly reduced compared to the CuSO_4_treatments alone, and the reductions were 36% and 41%, respectively, for KIN and BAP applications (Figure 6A). In the case of hydrogen peroxide (H_2_O_2_) accumulation, a drastic increase of 7- and 12-fold was recorded in the cotyledonary leaves treated with 80 and 160 µM CuSO_4_, respectively, compared to the control. When plants were exposed to exogenously applied KIN or BAP along with CuSO_4_, the content of H_2_O_2_ was found to be reduced significantly in the cotyledonary leaves (reduction of 62–79%) compared to the plants exposed to CuSO_4_ stress alone (Figure 6B).

A significant increase in lipid peroxidation as evidenced by increased accumulation of malondialdehyde (MDA) was observed in the cotyledonary leaves of *R. communis* subjected to Cu^2+^ stress. Compared to the control, the enhancement ranged from 4 to 6 folds in cotyledonary leaves exposed to 80 and 160 µM CuSO_4_. However, on the application of CuSO_4_ with KIN or BAP, the MDA accumulation was drastically reduced by 58–74% in the cotyledonary leaves of *R. communis* compared to those exposed to CuSO_4_ stress alone (Figure 6C).

### 2.7. Maintenance of Membrane Stability Index (MSI) in Response to Cytokinins

A significant reduction in membrane stability index (MSI) was observed in the cotyledonary leaves of *R. communis* subjected to Cu^2+^stress. On exposure to 80 and 160 µM CuSO_4_, the MSI was reduced to 57% and 67%, respectively, compared to the control. However, upon application of KIN or BAP along with CuSO_4_, the MSI was found to be restored to some extent in all four cases, and the reductions were only up to 19% compared to the MSI of control plants (Figure 6D).

### 2.8. Cytokinin-Mediated Enhancementsin Antioxidant Enzyme Activities

**Figure 7:**
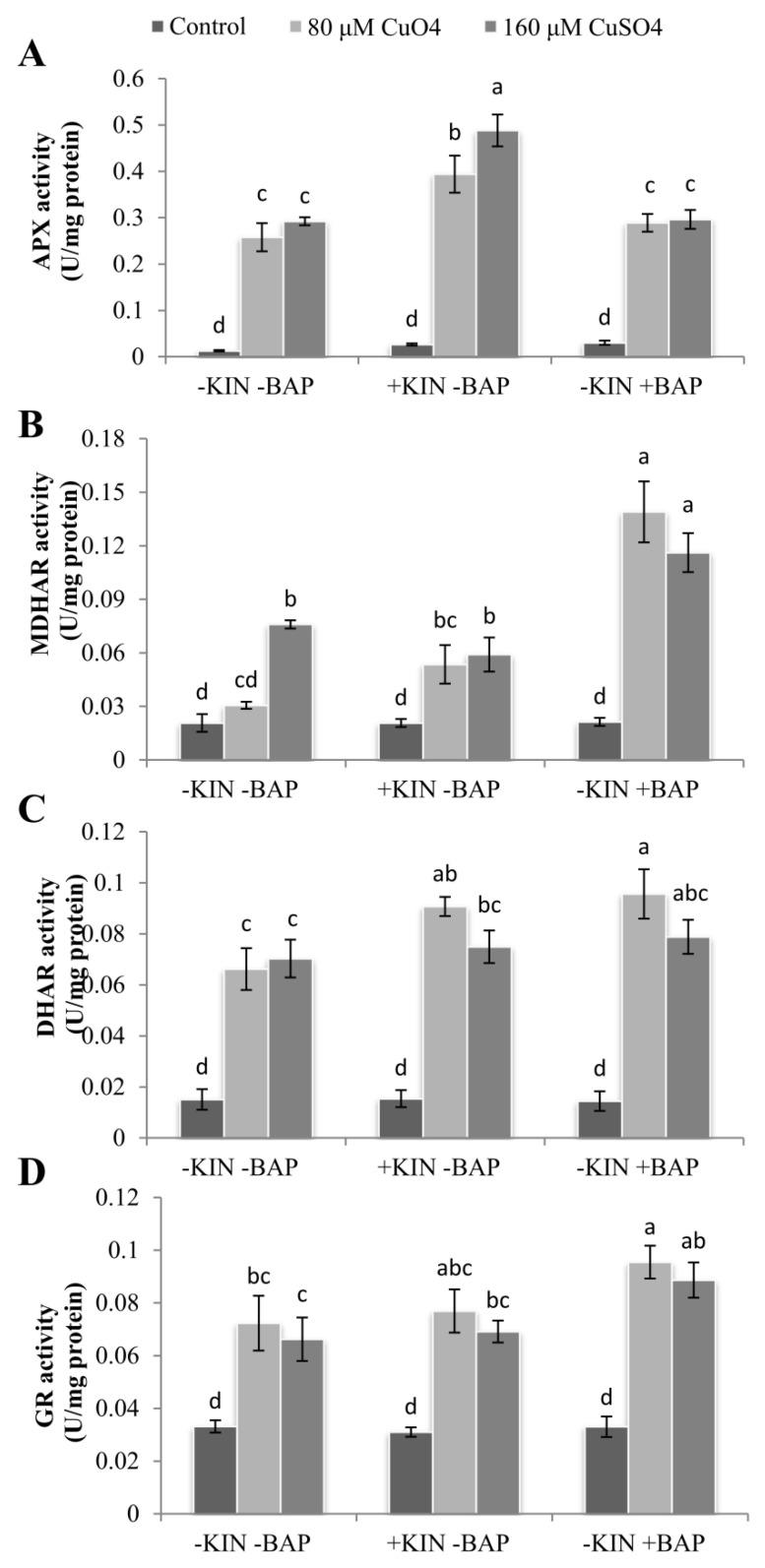
APX (**A**), MDHAR (**B**), DHAR (**C**), and GR (**D**) activities in the cotyledonary leaves of *R. communis* after 6 day of exposure to CuSO_4_ (80 and 160 µM) and cytokinin (KIN and BAP) treatments in Hoagland’s nutrient medium. Values are the mean ± SE of three independent experiments. Different alphabetical letters indicate significant differences between treatments according to one-way ANOVA (Duncan’s test *p* ≤ 0.05).

The activities of enzymatic antioxidants such as ascorbate peroxidase (APX), monodehydroascorbate reductase (MDHAR), dehydroascorbate reductase (DHAR), and glutathione reductase (GR) were significantly enhanced in the cotyledonary leaves of *R. communis* subjected to Cu^2+^stress (Figure 7). The enhancement in APX activity was 20 and 23 folds greater, respectively, for 80 and 160 µM CuSO_4_-treated cotyledonary leaves of plants compared to controls. The activity of APX was found to be significantly enhanced further with the application of KIN and BAP along with CuSO_4_compared with the cotyledonary leaves of *R. communis* exposed to Cu stress only, and the increase recorded was up to 67% (Figure 7A).

The enhancement in MDHAR activity was 1.5 and 3.7 folds higher in 80 and 160 µM CuSO_4_-treated cotyledonary leaves of *R. communis* compared to controls. In the case of DHAR and GR activities, CuSO_4_ treatment resulted in a 4.4–4.7- and 2–2.2-fold increase compared to control. Similar to APX activity, MDHAR, DHAR, and GR activities were also further enhanced upon the application of KIN and BAP along with CuSO_4_. The application of BAP enhanced the activities of MDHAR and DHAR to a maximum of 4.5 and 1.5 folds in cotyledonary leaves of *R. communis* compared plants subjected to CuSO_4_ alone. The enhancement in GR activities was the least in Cu-stressed cotyledonary leaves of plants upon exposure to KIN. In contrast, BAP application significantly enhanced GR activity in the cotyledonary leaves of plants subjected to Cu stress, especially in the case of 80 µM CuSO_4_, compared to the cotyledonary leaves of plants subjected to CuSO_4_ alone. This enhancement was found to be 4–6% and 32–34% in KIN- and BAP-treated plants, respectively (Figure 7B–D).

### 2.9. Influence of Cytokinins on Accumulation of Non-Enzymatic Antioxidants during Cu Stress

**Figure 8:**
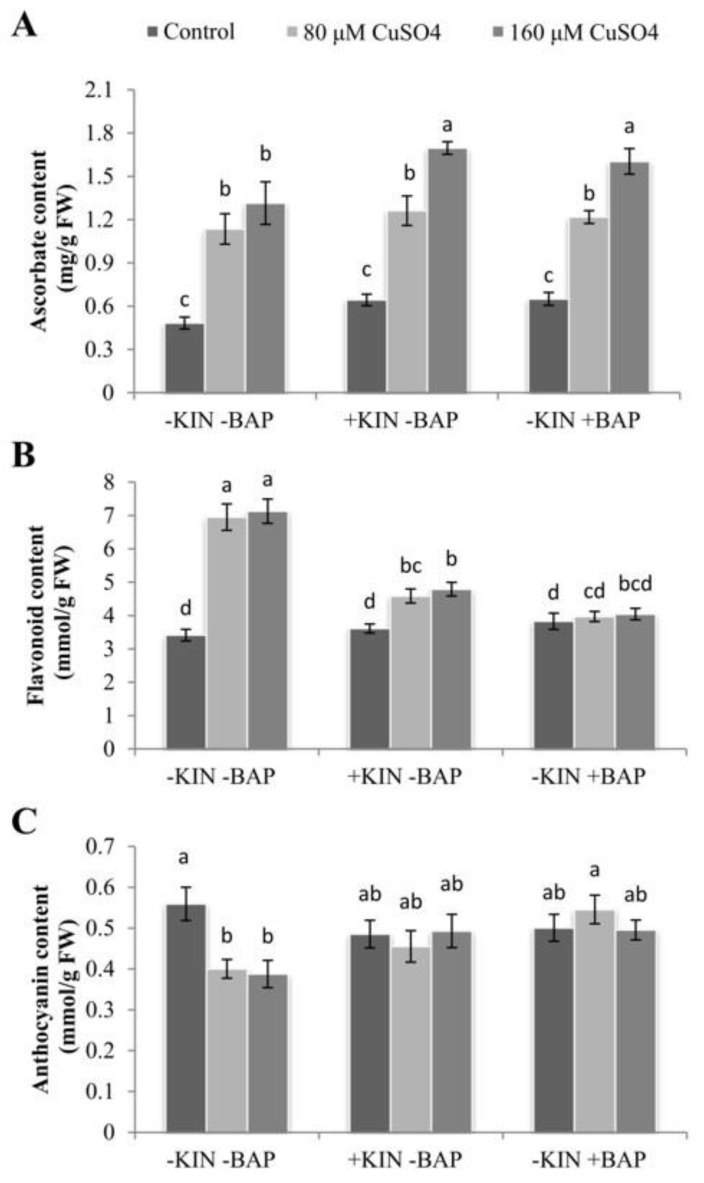
Ascorbate (**A**), flavonoid (**B**), and anthocyanin (**C**) contents in the cotyledonary leaves of *R. communis* after 6 day of exposure to CuSO_4_ (80 and 160 µM) and cytokinin (KIN and BAP) treatments in Hoagland’s nutrient medium. Values are the mean ± SE of three independent experiments. Different alphabetical letters indicate significant differences between treatments according to one-way ANOVA (Duncan’s test *p* ≤ 0.05).

Copper stress resulted in the enhanced accumulation of various antioxidants in the cotyledonary leaves of *R. communis* in the presence and absence of applied cytokinins (Figure 8). The increase in ascorbate (AsA) accumulation was 135% and 172% in 80 and 160 µM CuSO_4_ treatments compared to controls, respectively. Upon application of KIN or BAP, AsA accumulation was further increased (Figure 8A). Similarly, the accumulation of flavonoid contents was also found to be significantly enhanced upon exposure to Cu, and the enhancement exceeds 100% in both 80 and 160 µM CuSO_4_-treated cotyledonary leaves of *R. communis*. The application of KIN and BAP significantly reduced the accumulation of flavonoid contents. The reduction was found to the extent of 33–43% in both CuSO_4_ concentrations compared to the respective CuSO_4_ concentrations alone. Even though the accumulation of flavonoid contents was reduced in the cotyledonary leaves of plants subjected to Cu along with the applied cytokinins, the amount was higher than that of controls (Figure 8B).

In contrast to AsA and flavonoid contents, the accumulation of anthocyanin was significantly reduced on exposure to Cu, and the reduction was 28% and 31% in 80 and 160 µM CuSO_4_, respectively. However, upon the application of KIN and BAP, the anthocyanin content was seen to increase as compared to that of Cu stress alone. The enhancement in the accumulation of anthocyanin content ranged between 14–36% compared to the respective CuSO_4_ treatments, with the highest enhancement recorded in the BAP-treated plants (Figure 8C).

## 3. Discussion

Abiotic stresses, including the toxicity of metals, restrict the growth and development of plants [25]. Exposure to higher concentrations of CuSO_4_ in the growing medium resulted in decreased tissue water status in the cotyledonary leaves of *R. communis*. This reduction was associated with reduced water use efficiency and photosynthesis under abiotic stress conditions, including heavy metal stress [26]. According to Wu et al. [27], exogenous cytokinins can effectively alleviate photosynthetic damage in plants caused due to various abiotic stresses, thereby enhancing the water use efficiency under stress situations. Cytokinins prevent chloroplast disassembly and degradation of pigments, proteins, and nucleic acids during senescence, resulting in delayed senescence and prolonged photosynthesis in leaves [28]. The phenotype of *R. communis* especially that of the cotyledonary leaves, indicated that the application of cytokinins effectively alleviated Cu-induced alterations in the tissue water status and enhanced the tolerance potential.This would ensure prolonged photosynthetic functions in cotyledonary leaves even under Cu stress and would immensely help in seedling establishment.

Exposure to Cu stress reduced photosynthetic pigment composition and photosynthetic efficiency in the cotyledonary leaves of castor plants. Copper-induced chlorophyll degradation and reduced photosystem (PS) II efficiency in the cotyledonary leaves of *R. communis* were already reported in our previous studies [15,29]. The results indicated that the exogenous application of cytokinins ameliorated the negative effect of Cu on the photosynthetic pigment composition, which was evidenced by the increased level of chlorophyll and carotenoid contents in the cotyledonary leaves subjected to Cu stress along with cytokinins as compared to those subjected to Cu stress alone. Talla et al. [30] observed the cytokinin-induced upregulation of genes involved in the chlorophyll cycle and enhanced accumulation of the chlorophyll cycle intermediate, 7-hydroxymethyl chlorophyll, in rice seedlings. Therefore, it can be stated that exogenous cytokinins play significant roles in maintaining the photosynthetic pigments in plants subjected to diverse stress situations via the upregulation of genes involved in the synthesis of various intermediates in the chlorophyll cycle.

Heavy metal stress has been shown to result in the degradation of the thylakoid membrane protein complex, which results in the blockage of electron transfer and thereby diminishes photosystem activities [31,32]. The significant reduction in the PSI and PSII activities in the cotyledonary leaves of castor plants subjected to Cu stress was significantly nullified by the exogenous application of cytokinins, as evidenced by the enhancement in the photosystem activities in cytokinin-treated plants along with CuSO_4_. This enhancement in photosystem activities during cytokinin application was due to the enhanced expression of the genes encoding the chlorophyll binding proteins *Lhcb4* and *Lhcb6* in the light-harvesting complex (LHC) and oxygen-evolving enhancer gene *PsbO* and *PsbP* in the oxygen-evolving complex (OEC) [30]. This integrated networking of LHC and OEC genes resulted in the maintenance of the antenna size of PSII, underlining the efficient role of cytokinin in maintaining the photosynthetic functions even under severe stress conditions. 

In order to elucidate the susceptibility of PSII towards Cu stress in the cotyledonary leaves of *R. communis* and its enhanced tolerance upon exposure to cytokinins, the primary photochemistry was explored via chlorophyll *a* fluorescence. The structure and function of photosystems in plants exposed to different stresses can be studied by chlorophyll *a* fluorescence analysis. The increase in Fo during Cu toxicity indicates the lowering of energy trapping by PSII reaction centers resulting from the physical dissociation of PSII core LHC [33]. According to Gururani et al. [34], the increase in Fo due to heat stress can be mitigated by the exogenous application of abscisic acid (ABA), indicating the capability of this phytohormone to regulate the stability of the PSII complex. A significantly smaller increase in Fo observed in the cotyledonary leaves of *R. communis* subjected to Cu toxicity along with BAP reflects the stress-ameliorative function of BAP, and it was much better than that observed with KIN. The restoration of the flattened OJIP curve due to Cu stress by the application of BAP is yet further proof for the stress-relieving function of BAP, and again this feature was also much better than KIN.

A highly significant reduction in Fv/Fo in cotyledonary leaves of Cu-treated plants corresponds to the destabilization of the photosynthetic apparatus and the water-splitting complex on the donor side of PSII. The highly reduced number of electrons transferred by PSII (Sm) and the turnover number (N) indicate the reduced number of excited electrons, leading to the lowering of the PSII efficiency in cotyledonary leaves of plants subjected to Cu stress, which was restored upon application of BAP along with CuSO_4_. The drastic reduction in PI(abs) during Cu toxicity indicates the negative effect of metal ions on the efficiency and charge separation capability of PSII. The noticeable reduction in area on exposure to Cu stress indicates the reduction in the pool size of oxidized Q_A_and the inhibition of electron transport from RC to the quinone pool [35]. The exogenous application of cytokinins helps to regain the reduced values of these fluorescence parameters in the cotyledonary leaves of *R. communis* subjected to Cu stress. Cytokinin-induced improvements in these fluorescence parameters could be correlated with the enhanced number of active reaction centers and restoration of D1 protein due to antioxidation mechanisms supported by the antisenescing function of cytokinins [36].

The photosynthetic yield parameters, such as PHI(Po), PHI(Eo), and PSIo, denoting the proportion of energy absorbed by chlorophyll molecules associated with PSII, were susceptible to Cu stress, as evidenced by the decrease in the values of PHI(Po), PHI(Eo) and PSIo as compared to controls. However, BAP application along with CuSO_4_ enhanced the yield parameters, indicating the enhanced photosynthetic functions in *R. communis* under Cu toxicity. The restoration of these fluorescence parameters on the application of BAP along with CuSO_4_was in agreement with Wu et al. [28], wherein they observed that exogenous application of BAP effectively modulated photosynthetic efficiency in *Solanum melongena* plants under salinity stress. The role of cytokinins to overcome the high light stress-induced photo-damage in *Arabidopsis thaliana* plants was demonstrated by Cortleven et al. [37], wherein the authors proposed that cytokinin-deficient transgenic *A. thaliana* and cytokinin receptor mutants exposed to high light stress exhibited stronger photoinhibition and drastic decline in the quantum efficiency of PSII. These observations underline the significant role played by cytokinins in effectively modulating the photosynthetic functions of plants under different abiotic stresses.

The decrease in ABS/CSm due to Cu toxicity highlights the decrease in the energy absorptive potential of the reaction centers in a particular cross-sectional area in response to Cu stress. This may be either due to the reduction in the antenna size of PSII or the structural alteration in minor antenna LHC components of PSII, leading to the decline in energy absorption by chlorophyll molecules [38]. Although ABS/RC was increased upon exposure to Cu stress, the same was not reflected in ABS/CSm, as the distribution frequency of active reaction centers in a cross-sectional area was lower. The presence of fewer RC in a unit area would have forced the existing RC to harvest light energy to their maximum capacity. Even though ABS/RC was higher during Cu stress, the absorbed light energy was dissipated without being utilized for photochemistry, as represented by higher values of DIo/RC. The reduced TRo/CSm during Cu stress is related to the decreased density of the active reaction centers that eventually reduce the trapping efficiency [39]. The reduction in the number of active RCs in plants subjected to metal toxicity leads to the transformation of electron excitation energy to heat energy, ultimately culminating in photo-inhibition [40]. Therefore, it could be deduced that Cu stress results in the inactivation of the donor side of PSII due to the disorganization of the antenna molecules and reaction centers, attributed to the increased Cu stress effects in the cotyledonary leaves. However, at the same time, cytokinin application to the cotyledonary leaves of castor plants subjected to CuSO_4_ resulted in the enhancement of energy flux parameters. Similar to our results, a cytokinin-mediated enhancement in energy flux parameters in *Trigonella* seedlings subjected to Cd stress was observed by Bashri and co-workers [36]. This mitigation of photosystem damage in the cotyledonary leaves of *R. communis* during Cu stress was due to the efficient action of the antioxidation system influenced by cytokinins.

Stomatal closure has been shown to be one of the major limitations to photosynthesis under heavy metal stress in plants [41]. In the present study, the closure of stomata during exposure to Cu stress in the cotyledonary leaves of castor seedlings would largely contribute to the reduced photosynthetic functions in these leaves. This metal-induced stomatal closure can be correlated with the reduced tissue water status during Cu stress. It may be induced by the direct interaction of metal ions with the guard cells or as an early impact of metal toxicity in the roots [42]. However, upon exposure to cytokinins, the water use efficiency was enhanced, and the photosynthetic functions were restored, most significantly in BAP-treated seedlings. These results are in agreement with the observations of Nguyen et al. [43], wherein the authors proposed that the reduced level of this hormone in plants due to the detrimental effect of metal stress can be recovered by external supplementation of cytokinins to the natural levels, which in turn helps the plant to overcome the stress situation and achieve the recovery of photosynthesis.

The stress-mediated stomatal closure, decline in CO_2_ fixation and reduced PSII efficiency lead to photoinhibition and associated overproduction of ROS molecules [44]. The increased accumulation of MDA and ROS molecules and reduced MSI in the present study indicate the inefficiency in the transfer of absorbed energy to PSII RCs and the insufficiency of the antioxidative system necessary to scavenge the elevated ROS molecules. The enhanced activities of APX, MDHAR, DHAR, and GR accompanied by an enhanced level of AsA in the cotyledonary leaves exposed to Cu stress along with the exogenous application of cytokinins indicates the prominent role of KIN and BAP in castor seedlings under Cu stress in regulating ROS-induced oxidative stress, and thereby, in effectively modulating photosynthetic efficiency. Previously, studies have proved that applying a low concentration of phytohormones such as ABA and BAP confers metal stress tolerance via enhanced ROS scavenging by activating antioxidant activities [23,45]. Upon application of cytokinins, the AsA and anthocyanin contents were also increased, indicating the enhanced potential of the plant to negotiate the ROS-induced oxidative and photosystem damages. The enhanced AsA content upon the application of cytokinins might also contribute as an alternative electron donor for PSII, which reduces the photo-inactivation processes [46]. This resulted in the reduction of ROS accumulation in thylakoid membranes and improved photosynthesis in castor seedlings exposed to Cu stress along with applied cytokinins. 

The results of the present study revealed that the Cu stress-mediated negative effect on photosynthetic efficiency in cotyledonary leaves of *R. communis* was effectively alleviated by the exogenous application of cytokinins. It was also clear from the results that, of the two cytokinins used, BAP was most effective in maintaining photosynthesis in the cotyledonary leaves of *R. communis* subjected to Cu stress. Therefore, BAP can be very well considered as a membrane stabilizer and oxidative stress alleviator in plants under Cu stress.

## 4. Materials and Methods

### 4.1. Plant Material and Experimental Design

*Ricinus communis* seeds (TMV 5) were obtained from Tapioca and Castor Research Station (TCRS), Tamil Nadu Agricultural University, Salem, Tamil Nadu, India. After surface sterilization with 0.1% HgCl_2_, the seeds were germinated in solarized sand under controlled greenhouse conditions of temperature (25 ± 5°C), light intensity (12 h daylight in the range of 28–600 μmol/m^2^/s) and relative humidity (60 ± 2%). Subsequently, 10 d old seedlings were transplanted into glass bottles (9 × 5cm^2^) containing quarter strength modified Hoagland nutrient media [47]. The nutrient media was replaced on alternate days to provide a fresh dose of nutrient elements and to avoid algal growth in the medium.

### 4.2. Treatment with CuSO_4_ and Cytokinins

*R. communis* plants with a growth period of 30 d (after complete emergence of the first pair of primary leaves) were treated with varying levels of CuSO_4_ solution (80 and 160 µΜ) prepared in Hoagland nutrient medium for 6 day (the concentration and day of CuSO_4_ exposure were fixed based on our earlier studies, Sameena and Puthur [17]). The plants growing in quarter strength Hoagland solution were taken as the control plants. Stock solutions of kinetin (KIN) and 6-benzylaminopurine (BAP) were prepared in double-distilled water. The Cu-treated plants were supplemented with 15 µM each of KIN and BAP, prepared in 0.1% teepol as the surfactant, and applied as foliar spray (5 mLper plant). The selection of 15 µM as the most effective concentration for both KIN and BAP was based on a standardization study (Supplementary Table S1). The control plants were treated with teepol in water. The cotyledonary leaves were harvested at 6 day for further analysis. 

### 4.3. Measurement of Tissue Water Status

To calculate the moisture content percentage (*MC*%) and dry weight percentage (*DW*%), the fresh weight of the samples were recorded and then oven-dried at 100°C for 1 h, followed by lowering the temperature of the oven to 60°C. The samples were re-weighed at regular intervals until the weights became constant. *MC*% and *DW*% were calculated by using the following equation:

$$DW\%=\frac{Dry\;weight}{Fresh\;weight}\times 100$$

$$MC\%=\frac{Fresh\;weight-Dry\;weight}{Fresh\;weight}\times 100$$

The protocol proposed by Weatherley [48] was used to determine relative water content (*RWC*). After determining the fresh weight, small pieces of the samples were immersed in distilled water and kept in the dark for 12 h. Consequently, leaves were blotted; turgid weight was measured, followed by the dry weight measurements, and *RWC* was calculated by the following equation,

$$RWC=\frac{Fresh\;weight-Dry\;weight}{Turgid\;weight-Dry\;weight}\times 100$$

### 4.4. Estimation of Photosynthetic Pigment Composition

According to the method of Arnon [49] and Lichtenthaler and Wellburn [50], chlorophyll and carotenoid contents were estimated using 80% (*v*/*v*) acetone. 

### 4.5. Analysis of Photosystem Activities

For the analysis of photosystem (PS) I and II activities, the isolation of thylakoids and assay of photosynthetic electron transport activities of the isolated thylakoids were carried out according to the method of Mirshad and Puthur [51].

A total of 500 mg of fresh tissue was homogenized in 5 mL ice-cold isolation buffer (pH 7.8), containing 400 mM sucrose, 10 mM NaCl, and 20 mM tricine with pre-chilled mortar and pestle. The homogenate was filtered with 4-layered cheesecloth and then centrifuged at 4°C for 6 min at 5000 rpm. After centrifugation, the supernatant was discarded, and the pellet was suspended in 500 μM suspension buffer (pH 7.5) containing 100 mM sucrose, 10 mM NaCl, 20 mM HEPES [*N*-(2-hydroxyethyl)piperazine-*N*-(2-ethanesulphonic acid)], and 2 mM MgCl_2_. This thylakoid suspension was stored at 4°C and used for the analysis of thylakoid electron transport activities.

The polarographic measurements of thylakoid activities were carried out by a Clark-type oxygen electrode (DW1/AD, Hansatech, Norfolk, UK) along with a digital control box (OXYG1, Hansatech, Norfolk, UK). PSI and PSII activities were represented in nmol of O_2_ consumed (PSI)/evolved (PSII) min^−1^ mg^−1^ chlorophyll. For the measurement of PSI activity, stock solutions of 500 mM DCMU (3-(3,4-dichlorophenyl)-1,1-dimethylurea), 10 mM DCPIP (2,6-dichlorophenolindophenol), 500 mM ascorbate, 5 mM MV (methyl viologen) and 1 M NaN_3_ were prepared. The 2 mL reaction mixture containing 20 μL each of DCMU, DCPIP, ascorbate and MV, 10 μL NaN_3_, and 40 μL thylakoid extract was prepared in suspension buffer (pH 7.5). PSI activity was measured in terms of oxygen consumption by using DCPIP as an artificial electron donor and MV as an exogenous electron acceptor. To measure PSII activity, a stock solution of 50 mM PBQ (p-benzoquinone) was prepared. The 2 mL reaction mixture containing 20 mL PBQ and 40 μL thylakoid extract was prepared in suspension buffer (pH 7.5). PSII activity was measured in terms of oxygen evolution by using PBQ as an artificial electron acceptor. The chlorophyll content of the thylakoid extract was estimated according to Arnon [49].

### 4.6. Analysis of Chlorophyll a Fluorescence Parameters

Chlorophyll *a* fluorescence measurements were carried out using a Plant Efficiency Analyzer (Handy PEA, Hansatech Ltd., Norfolk, UK). The cotyledonary leaf samples were subjected to dark adaptation for 20 min using light exclusion clips on the leaf surface. Fluorescence measurements were recorded up to 1 s by illumination with a continuous red light (3000 µmol photons m^−2^ s^−1^, 650 nm) by an array of three light-emitting diodes focused on the leaf surface. The data obtained from Handy PEA were analyzed with the help of Biolyzer software (Bioenergetics Laboratory, University of Geneva, Geneva, Switzerland) [52]. Since the data obtained from Handy PEA were invalid for 6 day, the fluorescence analysis was carried out on 4 day of treatment. 

### 4.7. Analysis of Leaf Micromorphological Characters

The micromorphology of cotyledonary leaves was evaluated using a scanning electron microscope (SEM) after 6 day of Cu stress. The leaf cuttings of different treatments were fixed in 0.1 M sodium cacodylate buffer (pH 7.2) containing 2.5% glutaraldehyde for 5 min. The specimens were rinsed twice with distilled water and dehydrated by passing through an ascending acetone series. Dried samples were mounted onto aluminum stubs using double-sided adhesive conducting carbon tape. After gold-palladium coating, photomicrographs were taken using a field emission scanning electron microscope (FESEM, Carl-Zeiss, Gemini 300, Jena, Germany).

### 4.8. Analysis of ROS Molecules

The estimation of superoxide (O_2_^−^) content was carried out by the method of Doke [53], and calculations were done using the extinction coefficient of 12.3 mM^−1^cm^−1^. The estimation of hydrogen peroxide (H_2_O_2_) content was done by Junglee et al. [54], and hydrogen peroxide content was calculated by using the pure form of H_2_O_2_ as standard.

### 4.9. Estimation of Malondialdehyde Content

Malondialdehyde content was determined as per Heath and Packer [55], and calculations were done using its extinction coefficient of 155 mM^−1^cm^−1^.

### 4.10. Estimation of the Membrane Stability Index

The membrane stability index (MSI) was estimated according to Sairam et al. [56].

### 4.11. Assay of Enzymatic Antioxidant Activities

For the assay of ascorbate peroxidase (APX) activity, the enzyme extracts were prepared according to the method of Yin et al. [57]. The activity of APX was assayed as per Nakano and Asada [58], and 1 unit of enzyme activity was defined as the amount of enzyme required to oxidize 1 μmol of ascorbate min^−1^. Monodehydroascorbate reductase (MDHAR) activity was determined according to the method of Hossain et al. [59], and 1 unit of MDHAR activity was defined as the amount of enzyme required to oxidize 1 μmol of NADH min^−1^. Dehydroascorbate reductase (DHAR) activity was determined according to the method of Dalton et al. [60], and 1 unit of DHAR activity was defined as the amount of enzyme required to catalyze the formation of 1 μmol of ascorbate min^−1^. Glutathione reductase (GR) activity was assayed following Carlberg and Mannervik [61], and 1 unit of GR activity was defined as the amount of enzyme required to oxidize 1 μmol of NADPH min^−1^.

### 4.12. Estimation of Non-Enzymatic Antioxidants

Quantitative estimation of ascorbate (AsA) content was carried out by Chen and Wang [62] using L-ascorbic acid as the standard. Anthocyanin and flavonoid contents were estimated according to Mancinelli et al. [63] and Mirecki and Teramura [64], respectively. The anthocyanin and flavonoid contents were calculated using their molar extinction coefficients of 34.3 and 33 mM^−1^cm^−1^, respectively.

### 4.13. Statistical Analysis

Duncan’s test was used for the statistical analysis of the results at a 5% probability level. Data were subjected to one-way ANOVA using SPSS software 20.0. The data are an average observation from three independent experiments, each with three replicates representing mean ± standard error.

## 5. Conclusions

The exogenous application of cytokinins enabled castor plants to counteract Cu-induced oxidative stress and helped to re-establish the cellular redox status. Moreover, the efficient antioxidation systems, reduced ROS production and effective stomatal responses enhanced the photosynthetic performance of the cotyledonary leaves. They improved the Cu stress tolerance in castor plants, and this was highly significant when BAP was applied compared to KIN. Therefore, the exogenous application of BAP can be an efficient practice to improve the photosynthesis of plants subjected to Cu stress. Furthermore, developing transgenic plants with the potential to overproduce cytokinins could be a better strategy for successful phytoremediation processes.

## Figures and Tables

**Table 1:** Dry weight percentage (*DW*%), moisture content percentage (*MC*%) and relative water content (*RWC*) in the cotyledonary leaves of *R. communis* after 6 day of exposure to CuSO_4_ (80 and 160 μM) and cytokinin (KIN and BAP) treatments in Hoagland’s nutrient medium. Values are the mean ± SE of three independent experiments. Different alphabetical letters indicate significant differences between treatments according to one-way ANOVA (Duncan’s test *p* ≤ 0.05).

Treatments	*DW*%	*MC*%	*RWC*
Control	10.169 ± 0.508 ^d^	89.831 ± 4.492 ^a^	86.179 ± 4.309 ^b^
80 μM CuSO_4_	25.806 ± 1.290 ^b^	74.194 ± 3.709 ^b^	37.097 ± 1.855 ^e^
160 μM CuSO_4_	34.783 ± 1.739 ^a^	65.217 ± 3.261 ^c^	27.273 ± 1.364 ^f^
KIN	8.511± 0.426 ^e^	91.489 ± 4.574 ^a^	91.489 ± 4.574 ^ab^
80 μM CuSO_4_ + KIN	8.642 ± 0.432 ^de^	91.358 ± 4.568 ^a^	77.083 ± 3.854 ^c^
160 μM CuSO_4_ + KIN	16.667 ± 0.833 ^c^	83.333 ± 4.167 ^a^	49.180 ± 2.459 ^d^
BAP	8.421 ± 0.421 ^e^	91.579 ± 4.579 ^a^	93.548 ± 4.677 ^a^
80 μM CuSO_4_ + BAP	9.001 ± 0.450 ^de^	91.002 ± 4.550 ^a^	88.349 ± 4.417 ^ab^
160 μM CuSO_4_ + BAP	9.211 ± 0.461 ^de^	90.789 ± 4.539 ^a^	76.667 ± 3.833 ^c^

**Table 2:** Total chlorophyll and carotenoid contents in the cotyledonary leaves of *R. communis* after 6 day of exposure to CuSO_4_ (80 and 160 μM) and cytokinin (KIN and BAP) treatments in Hoagland’s nutrient medium. Values are the mean ± SE of three independent experiments. Different alphabetical letters indicate significant differences between treatments according to one-way ANOVA (Duncan’s test *p* ≤ 0.05).

Treatments	Total Chlorophyll Content (mg/g FW)	Carotenoids Content (mg/g FW)
Control	1.115 ± 0.011 ^cd^	0.508 ± 0.006 ^c^
80 µM CuSO_4_	0.804 ± 0.015 ^e^	0.248 ± 0.007 ^f^
160 µM CuSO_4_	0.684 ± 0.022 ^f^	0.213 ± 0.003 ^g^
KIN	1.301 ± 0.056 ^b^	0.559 ± 0.009 ^b^
80 µM CuSO_4_ + KIN	1.064 ± 0.036 ^d^	0.284 ± 0.003 ^e^
160 µM CuSO_4_ + KIN	1.075 ± 0.004 ^d^	0.385 ± 0.029 ^d^
BAP	1.477 ± 0.064 ^a^	0.503 ± 0.009 ^c^
80 µM CuSO_4_ + BAP	1.433 ± 0.021 ^a^	0.622 ± 0.011 ^a^
160 µM CuSO_4_ + BAP	1.209 ± 0.013 ^bc^	0.417 ± 0.002 ^d^

**Table 3:** The flourescence parameters showing phenomenological energy fluxes per cross-section approximated by maximal fluorescence Fm (CSm) and specific membrane model per a single reaction center (RC) in the cotyledonary leaves of *R. communis* for 4 day of exposure to CuSO_4_ (80 and 160 µM) and cytokinin (KIN and BAP) treatments in Hoagland’s nutrient medium. ABS/CSm & ABS/RC—absorption flux, TRo/CSm & TRo/RC—trapped energy flux, ETo/CSm & ETo/RC—electron transport flux and DIo/CSm & DIo/RC—dissipated energy flux. Values are the mean ± SE of three independent experiments. Different alphabetical letters indicate significant differences between treatments according to one-way ANOVA (Duncan’s test *p* ≤ 0.05).

Treatments	ABS/CSm	TRo/CSm	ETo/CSm	DIo/CSm	ABS/RC	TRo/RC	ETo/RC	DIo/RC
Control	2525.333 ± 36.448 ^ab^	2102.333 ± 29.237 ^a^	1020.667 ± 23.355 ^a^	423.0 ± 14.572 ^d^	2.338 ± 0.082 ^b^	1.946 ± 0.063 ^d^	0.944 ± 0.014 ^a^	0.392 ± 0.021 ^b^
80 μM CuSO_4_	1869.667 ± 233.326 ^c^	1251.333 ± 273.866 ^c^	334.333 ± 121.436 ^c^	618.333 ± 41.882 ^ab^	4.286 ± 0.569 ^b^	2.707 ± 0.069 ^a^	0.658 ± 0.140 ^b^	1.579 ± 0.552 ^b^
160 μM CuSO_4_	748.0 ± 160.706 ^d^	158.667 ± 71.966 ^d^	5.0 ± 4.0 ^d^	589.333 ± 91.758 ^abc^	19.089 ± 6.566 ^a^	2.872 ± 0.097 ^a^	0.070 ± 0.029 ^c^	16.217 ± 6.511 ^a^
KIN	2633.667 ± 52.616 ^a^	2147.667 ± 44.946 ^a^	973.333 ± 26.187 ^a^	486.0 ± 10.599 ^bcd^	2.586 ± 0.068 ^b^	2.109 ± 0.051 ^cd^	0.955 ± 0.017 ^a^	0.477 ± 0.018 ^b^
80 μM CuSO_4_ + KIN	2194.667 ± 76.667 ^bc^	1691.333 ± 70.333 ^b^	600.0 ± 18.0 ^b^	503.333 ± 6.333 ^bcd^	2.880 ± 0.033 ^b^	2.219 ± 0.011 ^c^	0.788 ± 0.013 ^ab^	0.662 ± 0.022 ^b^
160 μM CuSO_4_ + KIN	1027.0 ± 129.418 ^d^	373.0 ± 115.754 ^d^	29.0 ± 14.422 ^d^	654.0 ± 67.023 ^a^	8.438 ± 1.556 ^b^	2.702 ± 0.084 ^a^	0.186 ± 0.041 ^c^	5.736 ± 1.579 ^b^
BAP	2673.667 ± 13.220 ^a^	2184.667 ± 15.857 ^a^	995.667 ± 61.974 ^a^	489.0 ± 7.638 ^bcd^	2.482 ± 0.036 ^b^	2.028 ± 0.026 ^cd^	0.922 ± 0.041 ^a^	0.454 ± 0.013 ^b^
80 μM CuSO_4_ + BAP	2679.333 ± 46.998 ^a^	2211.333 ± 28.386 ^a^	1027.333 ± 12.333 ^a^	468.0 ± 18.717 ^cd^	2.515 ± 0.054 ^b^	2.076 ± 0.037 ^cd^	0.964 ± 0.014 ^a^	0.439 ± 0.019 ^b^
160 μM CuSO_4_ + BAP	2458.0 ± 75.020 ^ab^	1931.667 ± 86.822 ^ab^	649.333 ± 63.679 ^b^	526.333 ± 13.569 ^abcd^	3.065 ± 0.122 ^b^	2.404 ± 0.062 ^b^	0.803 ± 0.031 ^ab^	0.661 ± 0.059 ^b^

## Data Availability

The data presented in the present study are available from the corresponding author on reasonable request.

## Supplementary Materials

The supplementary Table S1 is available online at https://www.mdpi.com/article/10.3390/ijms222413349/s1.
