# Supplementary material for: 6-Benzylaminopurine Alleviates the Impact of Cu2+ Toxicity on Photosynthetic Performance of Ricinus communis L. Seedlings
Source: Int J Mol Sci. 2021 Dec 12;22(24):13349. doi: 10.3390/ijms222413349 (PMC8709281; doi:10.3390/ijms222413349)
Supplement: Supplementary file 1 [file ijms-22-13349-s001.zip › ijms-1485639-supplementary.pdf]

Table S1. Total chlorophyll, carotenoids and MDA contents analyzed on 6 d of treatment in the cotyledonary leaves of *R. communis* subjected to different concentrations of $CuSO_4$ (80 and 160 μM) and cytokinins (KIN and BAP in 5, 10, 15, 20 and 25 μM) in Hoagland's nutrient medium. Values are the mean ± SE of three independent experiments.

**Treatments**	**Total Chlorophyll content (mg/g FW)**	**Carotenoids content (mg/g FW)**	**MDA content (μmol/g FW)**
Control	1.289 ± 0.03	0.423 ± 0.02	14.366 ± 0.17
80 μM $CuSO_4$	0.816 ± 0.02	0.252 ± 0.01	65.591 ± 11.87
160 μM $CuSO_4$	0.705 ± 0.01	0.215 ± 0.01	95.430 ± 11.56
80 μM $CuSO_4$ + 5 μM KIN	0.627 ± 0.02	0.281 ± 0.01	40.0 ± 0.64
80 μM $CuSO_4$ + 10 μM KIN	0.598 ± 0.06	0.259 ± 0.03	36.532 ± 0.89
80 μM $CuSO_4$ + 15 μM KIN	0.973 ± 0.22	0.285 ± 0.01	30.269 ± 0.16
80 μM $CuSO_4$ + 20 μM KIN	1.443 ± 0.02	0.525 ± 0.01	28.145 ± 0.24
80 μM $CuSO_4$ + 25 μM KIN	1.919 ± 0.09	0.658 ± 0.02	31.532 ± 0.24
160 μM $CuSO_4$ + 5 μM KIN	0.929 ± 0.01	0.354 ± 0.01	38.105 ± 7.06
160 μM $CuSO_4$ + 10 μM KIN	0.972 ± 0.11	0.349 ± 0.04	31.854 ± 1.21
160 μM $CuSO_4$ + 15 μM KIN	1.055 ± 0.01	0.379 ± 0.01	26.427 ± 0.99
160 μM $CuSO_4$ + 20 μM KIN	1.407 ± 0.02	0.545 ± 0.01	41.835 ± 1.31
160 μM $CuSO_4$ + 25 μM KIN	0.711 ± 0.01	0.308 ± 0.01	35.806 ± 1.61
80 μM $CuSO_4$ + 5 μM BAP	0.761 ± 0.01	0.320 ± 0.01	28.387 ± 0.32
80 μM $CuSO_4$ + 10 μM BAP	0.925 ± 0.01	0.337 ± 0.01	25.484 ± 0.32
80 μM $CuSO_4$ + 15 μM BAP	1.269 ± 0.01	0.422 ± 0.03	20.322 ± 1.29
80 μM $CuSO_4$ + 20 μM BAP	1.346 ± 0.01	0.491 ± 0.01	22.270 ± 0.19
80 μM $CuSO_4$ + 25 μM BAP	1.127 ± 0.01	0.402 ± 0.01	50.269 ± 1.08
160 μM $CuSO_4$ + 5 μM BAP	0.755 ± 0.01	0.321 ± 0.01	27.016 ± 0.40
160 μM $CuSO_4$ + 10 μM BAP	1.036 ± 0.01	0.368 ± 0.02	26.434 ± 0.45
160 μM $CuSO_4$ + 15 μM BAP	1.589 ± 0.01	0.621 ± 0.02	25.081 ± 0.08
160 μM $CuSO_4$ + 20 μM BAP	1.131 ± 0.01	0.322 ± 0.01	28.226 ± 0.32
160 μM $CuSO_4$ + 25 μM BAP	0.984 ± 0.01	0.398 ± 0.01	23.871 ± 0.86
